# Supplementary material for: Physiological Ageing of the Lumbar Intervertebral Disc Based on Magnetic Resonance Imaging, a Systematic Literature Review
Source: Medicina (Kaunas). 2025 Aug 8;61(8):1430. doi: 10.3390/medicina61081430 (PMC12388827; doi:10.3390/medicina61081430)
Supplement: Supplementary file 1 [file medicina-61-01430-s001.zip › PRISMA_2020_flow_diagram.pdf]

PRISMA 2020 flow diagram for new systematic reviews which included searches of databases, registers and other sources

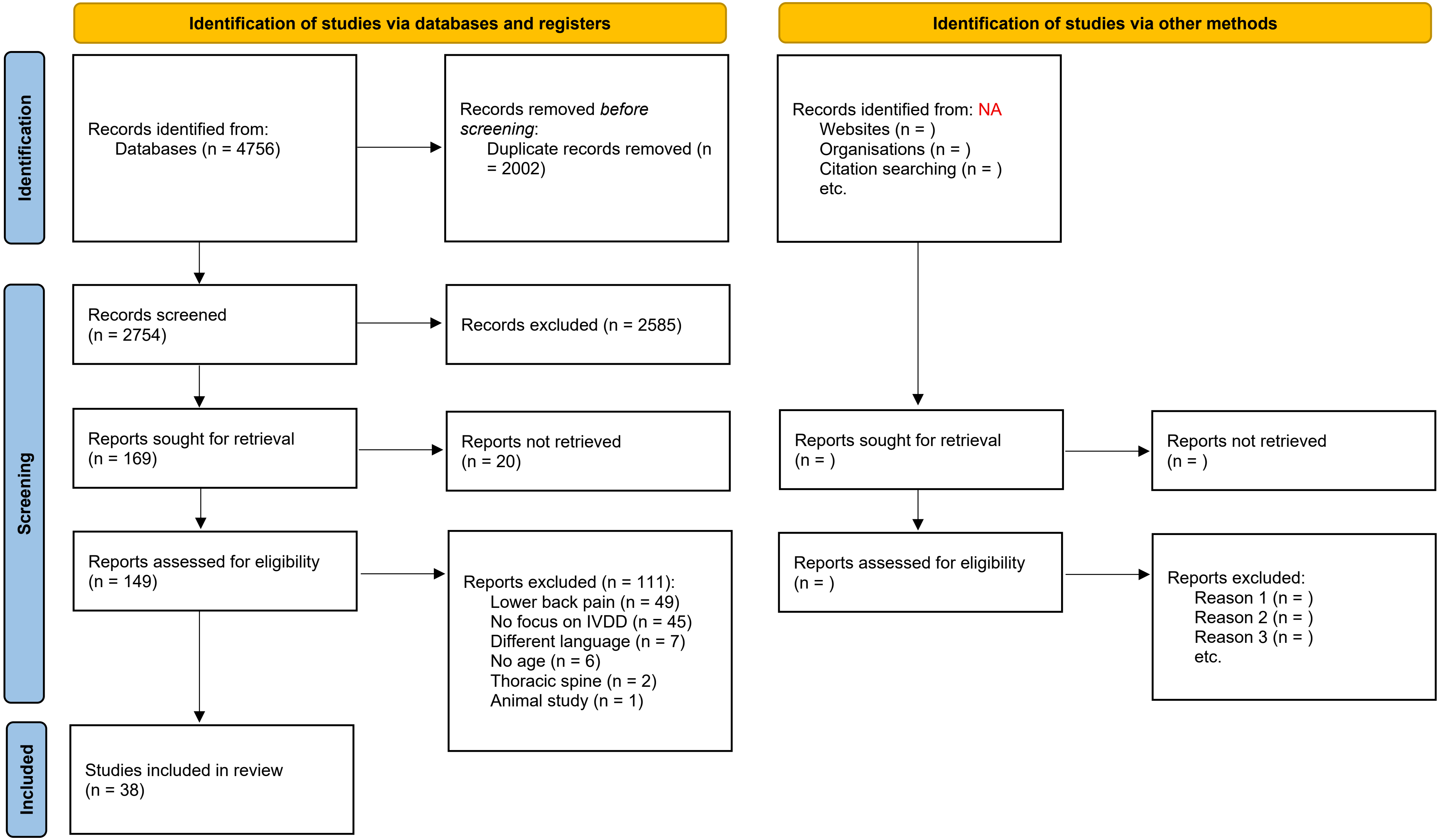

*Consider, if feasible to do so, reporting the number of records identified from each database or register searched (rather than the total number across all databases/registers).
**If automation tools were used, indicate how many records were excluded by a human and how many were excluded by automation tools.

From: Page MJ, McKenzie JE, Bossuyt PM, Boutron I, Hoffmann TC, Mulrow CD, et al. The PRISMA 2020 statement: an updated guideline for reporting systematic reviews. BMJ 2021;372:n71. doi: 10.1136/bmj.n71. For more information, visit: http://www.prisma-statement.org/
