# Supplementary material for: Physiological Ageing of the Lumbar Intervertebral Disc Based on Magnetic Resonance Imaging, a Systematic Literature Review
Source: Medicina (Kaunas). 2025 Aug 8;61(8):1430. doi: 10.3390/medicina61081430 (PMC12388827; doi:10.3390/medicina61081430)
Supplement: Supplementary file 1 [file medicina-61-01430-s001.zip › Supplemental File Tables.pdf]

Supplementary file S1

Table S1 Study characteristics part 1

Author (year)	Country	Design	Data collection	Level of evidence	Part of the lumbal spine scanned
Alkalay et al. (2018)	Israel	Non-Consecutive	Prospective	3b	L2/L3
Antoniou et al. (2004)	Canada	Non-Consecutive	Prospective	3b	L1/L2 - L5/S1
Ashinsky et al. (2020)	USA	Non-Consecutive	Retrospective	3b	L1/L2 - L5/S1
Auerbach et al. (2006)	USA	Non-Consecutive	Prospective	3b	L1/L2 - L5/S1
Bae et al. (2013)	USA	Non-Consecutive	Retrospective	3b	L1/L2 - L4/L5
Beattie et al. (2008)	USA	Non-Consecutive	Prospective	3b	L1/L2 - L5/S1
Berg-Johansen et al. (2019)	USA	Non-Consecutive	Retrospective	3b	L1/L2 - L5/S1
DeLucca et al. (2016)	USA	Non-Consecutive	Prospective	3b	L1/L2 - L5/S1
Ellingson et al. (2014)	USA	Non-Consecutive	Retrospective	3b	L4/L5
Filippi et al. (2013)	USA	Non-Consecutive	Prospective	3b	L1/L2 - L5/S1
Fyllos et al. (2018)	Greece	Non-Consecutive	Retrospective	3b	T12/L1 - L5/S1
Gübitz et al. (2018)	Germany	Non-Consecutive	Prospective	3b	L1/L2 - L5/S1
Haneder et al. (2014)	Germany	Non-Consecutive	Retrospective	3b	T12/L1 - L5/S1

Jaakko et al. (2006)	Finland	Non-Consecutive	Prospective	3b	L1/L2 - L5/S1
Johannessen et al. (2006)	USA	Non-Consecutive	Retrospective	3b	Not mentioned
Kolf et al. (2019)	Germany	Non-Consecutive	Prospective	3b	L2/L3 - L5/S1
Machino et al. (2022)	Japan	Non-Consecutive	Prospective	3b	T10/T11 - L5/S1
Matsumoto et al. (2012)	Japan	Non-Consecutive	Retrospective	3b	L1/L2 - L5/S1
Menezes-Reis et al. (2016)	Brazil	Non-Consecutive	Prospective	3b	L1/L2 - L5/S1
Moon et al. (2013)	USA	Exploratory cohort	Retrospective	2b	L1/L2 - L5/S1
Muftuler et al. (2014)	USA	Non-Consecutive	Retrospective	3b	L1/L2 - L5/S1
Nguyen et al. (2008)	USA	Non-Consecutive	Retrospective	3b	L1/L2 - L5/S1
Niu et al. (2011)	China	Non-Consecutive	Prospective	3b	L1/L2 - L5/S1
Pfirrmann et al. (2006)	Switzerland	Non-Consecutive	Prospective	3b	L1/L2 - L5/S1
Schleich et al. (2016)	Germany	Non-Consecutive	Prospective	3b	L1/L2 - L5/S1
Sharma et al. (2020)	USA	Non-Consecutive	Prospective	3b	T12/L1 - L5/S1
Shen et al. (2016)	China	Non-Consecutive	Prospective	3b	L1/L2 - L5/S1
Vadapalli et al. (2019)	India	Exploratory cohort	Prospective	2b	L1/L2 - L5/S1
Wang & Witschey et al. (2010)	USA	Non-Consecutive	Prospective	3b	L1/L2 - L5/S1

Wang et al. (2020)	USA	Non-Consecutive	Retrospective	3b	L4/L5 - L5/S1
Wang et al. 2017	China	Non-Consecutive	Prospective	3b	L1/L2 - L5/S1
Wei et al. (2022)	USA	Non-Consecutive	Prospective	3b	L1/L2 - L5/S1
Yang et al. (2017)	USA	Non-Consecutive	Retrospective	3b	L1/L2 - L5/S1
Yoon et al. (2016)	Republic of Korea	Non-Consecutive	Retrospective	3b	L1/L2 - L5/S1
Yu et al. (2014)	USA	Non-Consecutive	Prospective	3b	L1/L2 - L5/S1
Zhang et al. (2012)	China	Non-Consecutive	Retrospective	3b	L1/L2 - L5/S1
Zhang et al. (2014)	China	Non-Consecutive	Prospective	3b	L1/L2 - L5/S1
Zobel et al. (2012)	Italy	Non-Consecutive	Retrospective	3b	L1/L2 - L5/S1

Table S2 Study characteristics part 2

Author (year)	Study population	Age	MRI-Modalities	Tesla strength	Cadaver studies
Alkalay et al. (2018)	6 (not specified)	37-81 years	T2 relaxation and DWI	9.4T	Yes

Antoniou et al. (2004)	14 (8 men/ 6 women)	11-77 years (mean 48)	T2W + STE (Stimulated Echo sequence)	1.5T	Yes
Ashinsky et al. (2020)	7 (5 men/ 2 women)	60-90 years	T2-mapping + T2W	3.0T	Yes
Auerbach et al. (2006)	10 (5 men/ 5 women)	40-60 years	T2W + T1-rho	1.5T	No
Bae et al. (2013)	5 (4 men/ 1 woman)	Mean age 61 ±11 years	Proton density-weighted SE + UTE + T2W	3.0T	Yes
Beattie et al. (2008)	30 (16 men/ 14 women)	Mean age 36.7 ±12.8 years	T2W + DWI	3.0T	No
Berg-Johansen et al. (2019)	6 (4 men/ 2 women)	Mean age 58.5 ±5.2 years	T1ρ + UTE +T2W	3.0T	Yes
DeLucca et al. (2016)	9 (not specified)	42-75 years (mean 60.9 ±10.2)	T2W and 3D Flash MRI	7.0T	Yes
Ellingson et al. (2014)	18 (not specified)	21-71 years (mean 53.2 ±15.5)	T2 relaxation maps + T2W	3.0T	Yes
Filippi et al. (2013)	34 (15 men/ 19 women)	21-60 years	T1ρ + T2W	3.0T	No

Fyllos et al. (2018)	119 (59 men/ 60 women)	18-54 years (mean 33.08)	T2W	1.5T	No
Gübitz et al. (2018)	81 (40 men/ 41 women)	20-80 years	T1ρ	1.5T	No
Haneder et al. (2014)	55 (31 men/24 women)	21-60 years (mean 29.2 ±8.5)	23na-Imaging + T2W (T2-weighted)	3.0T	No
Jaakko et al. (2006)	20 (20 men)	40-56 years (mean 49)	T1 relaxation times after intravenous administration of Gd-DTPA-BMA + T2W	1.5T	No
Johannessen et al. (2006)	7 (not specified)	15-81 years (mean 51.6)	T1ρ + T2W	1.5T	Yes
Kolf et al. (2019)	40 (20 men/ 20 women	mean age 24.5 ±2.9 years	T1W + T2W + T2 relaxation maps	3.0T	No
Machino et al. (2022)	627 (307 men/ 320 women)	Mean age 49.6 ±16.5 years	T1W + T2W	1.5T	No
Matsumoto et al. (2012)	94 (48 men/ 46 women)	Mean age 48.0 ±13.4 years	T2W	1.5T	No
Menezes-Reis et al. (2016)	90 (42 men/ 48 women)	20-40 years (mean 27.1 ±4.8)	T1-rho and T2 relaxation mapping	1.5T	No

Moon et al. (2013)	17 (not specified)	Mean age 57.7 ±13.3 years	T1 mapping to construct FLASH 3D MRI (Fast low angle shot) sequence +T2W	7.0T	Mixed
Muftuler et al. (2014)	9 (6 men/3 women)	27-62 years (mean 45)	DCE (Dynamic contrast enhanced)-MRI and T2W	3.0T	No
Nguyen et al. (2008)	8 (not specified)	15-79 years (mean 51.8)	T2W and T1-rho	1.5T	Yes
Niu et al. (2011)	37 (25 men/ 12 women)	21-73 years (mean 40)	T2W + T2map + ADC	1.5T	No
Pfirrmann et al. (2006)	70 (37 men/ 33 women)	20-78 years	T2W + T1W	1.0T	No
Schleich et al. (2016)	48 (22 men/ 26 women)	21-49 years (mean 31 ±8)	T2W + gagCEST sequence (Glycosaminoglycan content sequence)	3.0T	No
Sharma et al. (2020)	101 (47 men/ 54 women)	25-35 years	T2 mapping +T2W	3.0T	No
Shen et al. (2016)	40 (19 men/ 21 women)	18.5-23.9 years (mean 24.35 ±1.8)	T2 relaxation mapping + DWI + DTI (Diffusion tensor imaging	1.5T	No
Vadapalli et al. (2019)	59 (35 men/ 24 women)	29-69 years	T2 relaxometry and DTI	3.0T	No

Wang & Witschey et al. (2010)	4 (not specified)	Mean age 47 years	MT (Magnetization transfer) + T2W	1.5T	No
Wang et al. (2020)	13 cadavers and 7 volunteers (not specified)	25-73 years (mean 36.9 ±10.9)	UTE (Ultrashort time echo sequence) + T2 relaxation mapping + T1ρ + T2W	3.0T	Mixed
Wang et al. 2017	93 (38 men/ 55 women)	20-76 years (mean 34.2 ±14.0)	T2W + T1-rho + Diffusion weighted imaging DWI	3.0T	No
Wei et al. (2022)	17 (8 men/ 9 women)	25-71 years (mean 43 ±16)	3D UTE-Adiab-T1ρ + T2W	3.0T	No
Yang et al. (2017)	65 (31 men/ 34 women)	22-59 years (mean 37.6)	T2W	1.5T	No
Yoon et al. (2016)	22 (8 men/ 14 women)	26-84 years (mean 55.5)	T1ρ + T2 mapping +T2W	3.0T	No
Yu et al. (2014)	9 (6 men/ 3 women)	27-62 years (mean 45)	DWI + T2W	3.0T	No
Zhang et al. (2012)	30 (19 men/ 11 women)	25-67 years (mean 46.8 ±16)	DTI + T2W	3.0T	No

Zhang et al. (2014)	109 (49 men/60 women)	20-59 years (mean 41 ±12)	DWI + T2W + T1W	3.0T	No
Zobel et al. (2012)	63 (34 men/29 women)	19-25 years (mean 22.95 ±1.8)	T1ρ + T2W	1.5T	No

Table S3 ROBINS-I risk of bias

Author (year)	Bias due to confounding	Bias in selection of participants into the study	Bias in classification of interventions	Bias due to deviations from intended interventions	Bias due to missing data	Bias in measurement of outcomes	Bias in selection of the reported result	Overall risk of Bias
Alkalay & David (2018)	Serious	Moderate	Low	Low	Low	Low	Low	Serious
Antoniou et al. (2004)	Low	Low	Low	Low	Low	Low	Low	Low
Ashinsky et al. (2020)	Low	Low	Low	Low	Low	Low	Low	Low
Auerbach et al. (2006)	Low	Low	Low	Low	Low	Low	Low	Low
Bae et al. (2013)	Low	Low	Low	Low	Low	Low	Low	Low
Beattie et al. (2008)	Low	Low	Low	Low	Moderate	Low	Low	Moderate
Berg-Johansen et al. (2019)	Moderate	Low	Low	Low	Low	Moderate	Low	Moderate
DeLucca et al. (2016)	Serious	Moderate	Low	Low	Low	Low	Low	Serious
Ellingson et al. (2014)	Serious	Moderate	Low	Low	Low	Low	Low	Serious

Filippi et al. (2013)	Low	Low	Low	Low	Low	Low	Low	Low
Fyllos et al. (2018)	Low	Moderate	Low	Low	Low	Low	Low	Moderate
Gübitz et al. (2018)	Low	Low	Low	Low	Low	Low	Moderate	Moderate
Haneder et al. (2014)	Low	Low	Low	Low	Low	Low	Low	Low
Jaakko et al. (2006)	Moderate	Low	Low	Low	Low	Low	Low	Moderate
Johannessen et al. (2006)	Moderate	Low	Low	Low	Low	Low	Low	Moderate
Kolf et al. (2019)	Low	Low	Low	Low	Low	Low	Low	Moderate
Machino et al. (2022)	Low	Low	Low	Low	Low	Low	Low	Low
Matsumoto et al. (2012)	Low	Moderate	Low	Low	Low	Low	Low	Moderate
Menezes-Reis et al. (2016)	Low	Low	Low	Low	Low	Low	Low	Low
Moon et al. (2013)	Serious	Moderate	Low	Low	Low	Low	Low	Serious
Muftuler et al. (2014)	Moderate	Low	Low	Low	Low	Low	Low	Moderate
Nguyen et al. (2008)	Low	Low	Low	Low	Low	Low	Low	Low
Niu et al. (2011)	Low	Low	Low	Low	Low	Low	Low	Low
Pfirrmann et al. (2006)	Low	Moderate	Low	Low	Low	Low	Low	Moderate
Schleich et al. (2016)	Low	Moderate	Low	Low	Low	Low	Low	Moderate
Sharma et al. (2020)	Low	Moderate	Low	Low	Low	Low	Low	Moderate

Shen et al. (2016)	Low	Low	Low	Low	Low	Low	Low	Low
Vadapalli et al. (2019)	Low	Low	Low	Low	Low	Low	Low	Low
Wang et al. (2020)	Serious	Low	Moderate	Low	Low	Low	Low	Serious
Wang et al. (2017)	Low	Low	Moderate	Low	Low	Low	Low	Moderate
Wang et al. (2010)	Serious	Moderate	Low	Low	Low	Low	Low	Serious
Wei et al. (2022)	Low	Moderate	Low	Low	Low	Low	Low	Moderate
Yang et al. (2017)	Low	Low	Low	Low	Low	Low	Low	Low
Yoon et al. (2016)	Low	Low	Low	Low	Low	Low	Low	Low
Yu et al. (2014)	Low	Low	Low	Low	Low	Low	Low	Low
Zhang et al. (2012)	Low	Serious	low	Low	Low	Low	Low	Serious
Zhang et al. (2014)	Low	Low	Low	Low	Low	Low	Low	Low
Zobel et al. (2012)	Low	Low	Low	Low	Low	Low	Low	Low

Table S4 Results and Summary/Conclusion

Author (year)	Results	Summary/Conclusion
Alkalay et al. (2018)	Grade II disks showed significantly higher mean T2, and ADC compared to grade III ((P < 0.01) and (P < 0.05) respectively) and grade IV ((P < 0.05) respectively) disks. No statically significant differences were found for either mean T2 or mean ADC between grade III and IV disks. Independent of region, donor age was negatively associated with mean ADC (P < 0.001), and positively associated with COV of ADC (P < 0.001) and T2 (P < 0.01). Cadaver age was negatively correlated with mean ADC (r = −0.58, P < 0.001). Compared to the AF, the NP exhibited a higher mean ADC (P < 0.05) and lower COV of ADC (P < 0.05) and of T2 (P < 0.01). COV of T2 was lower in the NP than in the AF (P < 0.01). In the NP, age was negatively correlated with mean of ADC (r = −0.73, P < 0.001) and T2 (r = −0.51, P < 0.01) and positively correlated with COV of ADC (r = 0.69, P < 0.001) and T2 (r = 0.29, P < 0.05).	For age 37-81 years ADC is significantly correlated to age, it decreases over time. A higher degenerative grade is associated with the decrease in mean T2 and ADC values. They also observed T2 values to moderately decrease between the 4th and 5th decade of life, increasing sharply thereafter between the 6th and 7th decade of life (T2 looks at water, initial loss of hydration in the NP, subsequently accumulation of water in the AF).
Antoniou et al. (2004)	The ADC in all three directions were significantly greater in grade 2 NP than the other degeneration-graded NP. The ADCs were greater in the NP than in the anterior and posterior AF of grade 2-degenerated discs only.	For age 11-80, the ADC decreased per age group (0-20;21-40;41-60;61-80) in the NP and anterior AF, not the posterior AF. Furthermore, moderate positive

	The ADCs in the x, y, and z directions decreased with age in the NP and in the anterior AF, except for the ADCx (Age groups 0-20; 21-40; 41-60; 61-80). Low positive correlations were found between each of the ADCs and the whole disc water content. In the subregion analysis, in the NP, moderate positive correlations were found between each of the ADCs and water content. Similar patterns were observed when the ADCs were correlated to the GAG content. No correlations were observed between each of the ADCs and the total collagen and total denatured collagen contents in the whole disc. No correlations were observed in the whole disc and each disc area between the ADCs and the total protein content.	correlations between water + GAG and the ADC in the NP were found for the entire age group. No correlation between ADC and collagen content was found.
Ashinsky et al. (2020)	With increasing disc Pfirrmann grade, T2 relaxation times in the NP decreased, with significant differences observed between grade 2 discs and grades 3 and 4 discs. In contrast, AF T2 relaxation times increased with increasing Pfirrmann grade, with no significant differences detected between any of the groups (Pfirrmann). NP T2 was positively correlated, and AF T2 negatively correlated with disc height.	For age 60-90 in this study, T2 relaxation times decreased in the NP with increased Pfirrmann and T2 relaxation time. Indicating a loss of GAG from the NP.

Auerbach et al. (2006)	The T1ρ values of lower discs were significantly lower than higher discs (P<0,01). T1ρ values correlate with the Pfirrmann grade (r=-0.51, P<0,01).	T1ρ correlates negatively with Pffirmann grade for the age of 40-60. T1ρ was significantly lower in lower discs than higher discs.
Bae et al. (2013)	Logistic regression of data from proton density–weighted SE imaging suggested that the presence of disk tissue in the sample was a strong predictor of the presence of signal intensity (P < .001), whereas the presence of uncalcified CEP (P = .2) and calcified CEP (P = .8) were not. Conversely, for data from ultrashort TE MR imaging, logistic regression suggested that the presence of uncalcified CEP (P = .023) and calcified CEP (P = .007) were strong predictors of the presence of signal intensity at ultrashort TE MR imaging, whereas the disk was not a strong predictor (P = .7).	UTE MR imaging enables direct visualization of CEPs, appearing as a bilaminar structure with a thick upper layer with an intermediate signal and a thinner lower layer with high signal intensity in the age group 61+/-11yrs.

Beattie et al. (2008)	The SEM ranged from 0.004 to 0.026*10^3 mm2/s, with the width of the 95% CIs ranging from 0.016 to 0.102*10^3 mm2/s. The mean ADC for all subjects at all levels was 1.83*10^3 mm2/s (SD, 0.21). There were no significant differences between the initial and follow-up mean ADC values.	The ADC has excellent inter and intra-rater reliability. IVDs with signs of degeneration had a higher between scan variability than non-degenerated discs. This is likely due to more variability in internal pressure of degenerated discs resulting in inconsistent diffusion rates. This observation is intriguing and supports the theory that diffusion of water in degenerative discs is extremely sensitive to excessive or prolonged loading, which, in turn, may act to propagate further degeneration and symptoms. Intermediate and hypointense NPs have lower ADCs, suggesting reduced water diffusion is associated with these T2 signals.
Berg-Johansen et al. (2019)	UTE-derived CEP thickness maps and disc T1ρ maps varied with Pfirrmann grade, with degenerated discs exhibiting poorly-delineated CEPs with point defects and lower thickness values. The standard deviation of T1ρ was significantly lower for Pfirrmann grade 4 than Pfirrmann grade 3 ($p < 0.05$). The mean T1ρ also decreased with Pfirrmann grade, although the difference was not significant. The CV CEP	For age 58.5 (+/-5.2)the variability in both CEP thickness and T1ρ were significantly related to degeneration grade (Pffirmann). Indicating that spatial heterogeneity in the CEP and disc properties are more indicative of degeneration.

	thickness was significantly higher for Pfirrmann grade 4 than Pfirrmann grade 3 ($p < 0.05$), while the mean CEP thickness was unaffected by Pfirrmann grade. The standard deviation of T1ρ and CV CEP thickness had a significant negative correlation.	
DeLucca et al. (2016)	CEP thickness was greater in anterior and posterior locations than in the center of the disc, concave shape. While center thickness did not correlate with age ($r = -0.04$, $p > 0.05$), the A/P thickness ($r = -0.40$, $p < 0.01$) and average thickness ($r = -0.44$, $p < 0.01$) both decreased with age. CEP axial area ($r = -0.37$, $p < 0.05$) and lateral width ($r = -0.42$, $p < 0.01$) both decreased with age while A-P width ($r = -0.25$, $p > 0.05$) did not correlate with age. No CEP geometry parameters correlated with degeneration measured by T2 or Pfirrmann grade.	For the age 42–75 years CEP morphology changes in a linear correlation with age, the anterior and posterior thickness decrease as well as the axial area and lateral width. A decreased axial area possibly leads to altered transport into the IVD as there is just less surface area for diffusion. There was no correlation between CEP parameters and disc degeneration.
Ellingson et al. (2014)	Pfirrmann grade was significantly correlated with average T2* relaxation time (ranged from 9.1 to 91.6 ms) in the NP ($r = -0.891$; $p < 0.001$) and iAF (Inner lateral annulus fibrosis) ($r = -0.749$; $p < 0.001$). The mean T2* relaxation time was positively correlated with s-GAG normalized by dry weight in all five regions of interest. The absolute changes become less for the more degenerative tissues, while still maintaining the observed	T2* relaxation time at all 5 locations (NP, iAF, oAF, aAF, pAF) were significantly and positively correlated with the s-GAG (measure of PG) content, especially in the NP, for age 21-71. There is increased decrease of T2* in early stages and decreased with higher degeneration (Pfirrmann).

	linear relationship. In other words, the T2* relaxation time drastically decreases early in the degeneration process, similar to the proteoglycan content, then slows down during later stages.	
Filippi et al. (2013)	There was a statistically significant moderate negative correlation between T1ρ values and subject age at the L1-2 ($r = 0.585$), L2-3 ($r = 0.853$), L3-4 ($r = 0.793$), and L4-5 levels ($r = 0.750$) ($p < 0.001$) and at the L5-S1 level ($r = 0.522$) ($p < 0.01$). There was a statistically significant difference ($p < 0.01$) in the T1ρ values between all the age groups sampled, and this difference was maintained between individual groups with the exception of the age groups for 40– 49 years and 50–59 years.There was a statistically significant ($p < 0.01$) difference for T1ρ values between lower-grade disks (Pfirrmann grades 1–3) but not between higher-grade disks (Pfirrmann grades 4 and 5). The kappa statistic of interobserver reliability of T1ρ measurements was excellent, at 0.934. No male/female difference.	There was a decrease in T1ρ from age 20-49 after which there was no significant difference between age group 40-49 and 50-59. Theory for this is that proteoglycan loss (Pfirmann 1-3) precedes disc desiccation (Pfirrmann 4-5) after which normal signal intensity is lost.
Fyllos et al. (2018)	The overall regression model was significant ($p < 0.001$) with $R2 = 0.598$ for DHI, $R2 = 0.604$ for Dabbs, $R2 = 0.324$ for Farfan, $R2 = 0.198$ for disc convexity index and $R2 = 0.669$ for mean disc height	This study created baseline values for disc height and volume in a healthy population aged 18-54yrs, not considering variation per decade.

Gübitz et al. (2018)	There is a significant decrease in the T1ρ relaxation times between L1/2 and L5/S1 ($P = 0.0013$). The T1ρ relaxation time was lower in the AF and higher in the NP. In relation to the three age groups (A–C), significant differences were detected between groups A and C ($P = 0.0008$) and between groups B and C ($P = 0.0149$) using a linear mixed model with inclusion of lumbar level, age group, and disc part.	Age showed a significant and uniform impact on T1ρ relaxation times. This suggests proteoglycan loss in the disc over time. From age 20-80. With increased degeneration after the age of 59. Increased degeneration with more caudal discs.
Haneder et al. (2014)	Normal distribution of 23Na in healthy volunteers. 23Na norm was significantly reduced in IVDs with a high Pfirrmann score of 4 (23Na-norm=0.89+/-0.2) and 5 23Na-norm=0.85+/-0.4) compared with Pfirrmann 1 to 3 (23Na-norm=1.44+/-0.2; 23Na-norm=1.40+/-0.1; 23Na-norm=1.32+/-0.1). There is no to weak correlation between 23Na-MRI and age in healthy volunteers ($0.007<R2 <0.284$).	23Na-MRI is a potential non-invasive biomarker which correlates to the degree of degeneration (Pfirrmann) for the age 29.2 (+/-8.5). There is no correlation between age and 23Na-MRI for this age group, suggesting that there is no loss of glycosaminoglycan during this time.
Jaakko et al. (2006)	Measured T1 times before and 90 minutes after Gd-DTPA-DMA injection vs. Pfirrmann's degeneration grade (mean ± 1 SD). Both pre- and postcontrast measurements show a decreasing correlation with the degeneration grade. The difference between T1 and T1Gd is larger in the more-degenerated discs (not in grade II, III). A positive trend was	The results suggest that T1 relaxation time can be used to quantify contrast enhancement after contrast injection in degenerated discs. The nucleus pulposus of degenerated (Pfirrmann grading) discs enhanced more

	observed between the change in the T1 relaxation rate and the degeneration grading.	rapidly and more intensively than that of normal discs in subjects aged 40-56.
Johannessen et al. (2006)	There was a strong correlation between T1ρ and degenerative grade ($r = -0.76$, $P < 0.01$). T1ρ was strongly correlated with sulfated-glycosaminoglycan per wet weight ($r = 0.70$, $P < 0.01$), and was moderately correlated with sulfated-glycosaminoglycan per dry weight ($r = 0.67$, $P < 0.01$) and water content ($r = 0.58$, $P < 0.05$). There was a strong correlation between T1ρ and age ($r = -0.76$, $P < 0.01$).	T1ρ is directly correlated with PG of the nucleus pulposus. This has a strong correlation with age and indicates the loss of PG in the NP from 15-81yrs, not taking into account variation per decade. Future research needs to focus on the AF and articular cartilage.
Kolf et al. (2019)	There was a difference however between high and lower lumbar levels and between outer and inner zones of the discs, T2 value was highest in the NP and decreased further outwards. No significant difference between men and women.	This study provides baseline normative data on T2* values for asymptomatic, non-degenerated human IVD in the lumbar spine (Pffirmann grade 1). For this age group (21-34yrs) T2 values are low at the AF and increase towards the NP, suggesting high water content in the NP and more collagen in the AF. Lower lumbar discs are more affected than higher discs.

Machino et al. (2022)	The intervertebral disc height at the anterior edge, center and posterior edge gradually increased from T10/T11 to L4/L5 or L5/S1 in all age groups. Disc index gradually decreased with increasing age in middle-aged and elderly individuals in males, with the L4/L5 level being the most prominent. In both the genders along with disc degeneration grade, the disk index from L2/L3 to L5/S1 levels also decreased.	Lumbar disc height narrowing progresses with age and is correlated with the progression of degeneration of the disc. Furthermore, disk height increases from higher to lower lumbar levels in all age groups.
Matsumoto et al. (2012)	A decrease in disc signal intensity in the lumbar spine was significantly associated with an increase in age [odds ratio (OR) 4.2; 95 % confidence interval (CI) 1.2–14.9; p = 0.024]. Posterior disc protrusion in the lumbar spine was significantly associated with increased age (OR 7.9; 95 % CI 2.0–32.2; p = 0.004).	With the progression of age there is a reduction of LVD signal intensity on T2-weighted images. Furthermore, cervical and lumbar degeneration often occurs in tandem. Finally, degenerative signs are seen at all ages, frequency increases with age (48 +/- 11yrs). More degenerative signs at more caudal discs.
Menezes-Reis et al. (2016)	The cranial and caudal segments of the lumbar spine, namely L1L2 and L5S1, respectively, had the lowest relaxation time values, while the highest values were observed for the L3L4 central disc segments. A similar trend was observed for the IVD volumes. There was no significant difference between men and women for either relaxometry map (T2,	For age 20-40 years T2 relaxometry identified gradual disc dehydration related to aging in the first two decades of adulthood. There was no correlation with age for T1ρ.

	P=0.37; T1ρ, P=0.97). The relaxation times of the NP were higher than the anterior and PAF both T2 and T1ρ.	
Moon et al. (2013)	The mean CEP thickness was 0.77 ± 0.24, no significant CEP thickness was observed across disc levels and locations. There were significant effects of anterior–posterior location on CEP thickness, where the minimum thickness was at the center of the disc (0.54 ± 0.12 mm, averaged across all lumbar levels). The thickness at the center was 23 % less than the 0.5 cm anterior–posterior location and 44 % less than the 1.0 cm anterior–posterior location ($p < 0.005$), resulting in a "V" shaped pattern across the disc.	FLASH 3D MRI is good at distinguishing and creating a 3D model of the CEP. For the age 57 yrs (+/-SD) the cep has a V shape. CEP thickness is not related to disc level. Potential tool for non-invasive assessment and quantification of disc health.
Muftuler et al. (2014)	No DCE enhancement was seen in the NP of any disc. The avascular cartilaginous endplates show markedly different contrast agent uptake and washout characteristic than subchondral bone, with a lower enhancement peak and no noticeable washout. There is higher DCE enhancement in grade 4/5 Pfirrmann discs than in 2/3 grade discs. Significantly higher enhancement in caudal endplates.	DCE increases with higher degeneration (Pfirrmann). No DCE was seen in the NP of any disc, meaning no fluid transport. The increased flow in higher degenerated discs (Pfirrmann 4-5) seems contradictive but could indicate the leakiness of the CEP and overall loss of hydration and matrix proteins for healthy volunteers age 21-60.
Nguyen et al. (2008)	The T1ρ relaxation time was strongly correlated with the degenerative grade ($r = -0.83$, $p < 0.05$) and age ($r = -0.84$, $p < 0.05$), and it was	T1ρ is correlated with water and proteoglycan content of the intervertebral disc. T1ρ-weighted was also correlated

	significantly higher for the fifteen non-degenerated discs than it was for the eighteen degenerated discs. A positive linear correlation was observed between the T1ρ relaxation time on the images of the nucleus pulposus and the swelling pressure ($r = 0.59$), glycosaminoglycan content per dry weight ($r = 0.69$), glycosaminoglycan per wet weight ($r = 0.49$), and water content ($r = 0.53$).	with age and degenerative grade (Pfirrmann), indicating increased degeneration with age and decreased water and PG content of the NP with age from 21-80yrs.
Niu et al. (2011)	A well-defined asymptomatic population of volunteers was recruited and divided into 5 groups according to age. Both T2 and ADC values in their lumbar disks were found to correlate with age. Linear regression analysis revealed that T2 exhibited a more significant inverse correlation with age ($r = -0.77$, $P < .01$) than ADC ($r = -0.37$, $P < .01$).	For the age 21-73 ADC and T2 showed an inverse correlation with age, the correlation with T2 was stronger than ADC. The inverse correlation with T2 suggests that there is decreasing glycosaminoglycan and water content in the IVD. Looking at age groups, there seems to be a drop in ADC after age 50 however no statistical testing on this has been performed for this.
Pfirrmann et al. (2006)	Multilevel regression analysis showed that disc height exhibits a significant ($p<0.001$) positive correlation with the disc level. Disc degeneration resulted in an overall decrease of disc height ($p<0.01$). Increasing age was positively correlated ($p<0.01$) with disc height (i.e., the older the subject, the higher the disc), although not reaching	For age 20-78, disc degeneration results in a decrease of disc height and volume, as well as a less convex disc shape, by doing so a linear relation with age is assumed. In the absence of disc degeneration age tends to result in

	statistical significance. Multilevel regression analysis showed that disc degeneration is a significant predictor of disc volume ($p<0.001$). There was also a strong positive correlation with age($p<0.01$), and body height ($p<0.001$).	an inverse relationship on disc height, volume, and shape for this age group.
Schleich et al. (2016)	Non-degenerative IVDs (Pfirrmann score 1–2) showed significantly higher gagCEST values in NP and AF compared with degenerative IVDs (Pfirrmann score 3–5). The MTRasym values were higher in non-degenerative lumbar IVDs (Pfirrmann 1–2) compared with degenerative lumbar discs (Pfirrmann 3–5; 2.92% ± 1.42% vs. 0.78% ± 1.38%; $P < 0.0001$). The MTRasym values of NP were significantly higher in normal appearing discs compared with herniated IVDs (2.83% ± 1.52% vs. 1.55% ± 1.61%; $P < 0.0001$). We found a significant negative correlation between gagCEST values and the graduation of disc herniation ($r = -0.372$; $P < 0.0001$). We could demonstrate a significant correlation between age and morphological Pfirrmann classification ($r = 0.3175$; $P < 0.0001$), as well as between age and CTF classification ($r = 0.2476$; $P < 0.0001$).	For age 21-49 non-degenerative IVDs (Pfirrmann score 1–2) showed significantly higher gagCEST values in NP and AF compared with degenerative IVDs (Pfirrmann score 3–5). As well as linear relationship with Pfirrmann and CTF with age.

Sharma et al. (2020)	In total 606IVDs were scanned. 493 discs Pffirmann <2 were considered 'healthy' of which 489 were grade 2. Mean T2 and T2np values for all IVDs were 115.5 ± 24.6 ms and 131.5± 30.0 ms, respectively. IVDs graded healthy (Pfirrmann grades 1 and 2) had higher T2 (121.1 ± 22.5 ms) and T2np (138.6 ± 26.8 ms) values compared with corresponding T2 and T2np values of 91.5 ± 18.6 ms and 101.0 ± 23.3 ms, respectively, for degenerated IVDs ($P < .001$ for both). Intrasubject variability measures (I and Inp) were significantly lower than any of the corresponding intersubject variability measures ($P < .001$ for all regions).	The results suggest that that other healthy IVDs within an individual, if available, are likely to provide the most optimal basis of the definition of normal against which a given IVD should be compared due to high intersubject variability. At this age group Pffirmann grade 2 is most common and almost no 'healthy' discs are seen according to this grading system.
Shen et al. (2016)	ADC values decreases per anatomical level, and the mean values of FA and T2 also correlate with anatomical levels (increasing). The highest ADC values of the IVD appear in the NP/centre and decline towards the periphery, the FA and T2 values were lowest at the center of NPs and increased towards the periphery ($P < 0.05$).	From age 18-24, the ADC is high at the centre/NP and decrease towards the periphery, for FA and T2 it is low in the centre and increases to the periphery. This suggests high PG and water content in the centre and towards the periphery more collagen and less water. Higher ADC at lower lumbar levels indicates more free water and possibly early stage IVDD at this age.
Vadapalli et al. (2019)	Among the healthy controls, subgroup A had a higher T2 and AF/NP ratio than subgroup B; however, the T2 AF was not significantly different.	For the age 29-69 a mean decrease in FA values in subgroups A (<30), B (30-50), and C (>50), with

	Subgroups A and B had a higher T2 and AF/NP ratio than subgroup C but lower NP and AF values. AF/NP values were strongly correlated with age and with the T2 map (ms) in the healthy controls. AF/NP was strongly correlated with increasing age in the healthy controls. The control group presented mean T2 values >120 ms in subgroup A, 90–100 ms in subgroup B and approximately 70 ms in subgroup C (p<0.001).	progressive degeneration was observed. Furthermore, subgroups A had a higher T2 and AF/NP than subgroup B and subgroup A and B had a higher T2 and AF/NP ratio than subgroup C although the FA was lower. Indicating loss of water and loss of texture/integrity as well as a more heterogenous disk with age.
Wang et al. (2010)	NP MTR vs. Pfirrmann grade data yields a moderate but significant Pearson correlation coefficient of 0.652 at a significance level of P < 0.01, as well as a moderate but significant Spearman's rank correlation coefficient of 0.568 at a significance level of P < 0.01.	For age 47 the NP MTR is correlated with Pffirmann grading, the increased of the MTR is likely due to an increase in collagen density in the NP (potentially due to water loss).
Wang et al. (2020)	Age is inversely associated with both mean T1ρ values in the NP (r = −.72, P < .001) and mean T2* values in the central CEP (r = −.45, P = .032). After splitting the discs into three similarly sized groups based on age (n = 8, 7, 8), there was a significant interaction between the effects of age and CEP T2* values on NP T1ρ values (P = .045), suggesting that the effect of CEP T2* values on NP T1ρ values depends on age. Also, NP T1ρ values were significantly correlated with CEP T2*	High collagen and low water content of the CEP impacts NP cell viability and gene expression in the earlier stages of degeneration age <50. In other words, deficits in CEP composition as indicate by low T2* values, associate with more severe disc degeneration during the mild-to-moderate stages in younger individuals. UTE T2 has a very good repeatability.

	values (r = .71, P = .047), but only in the youngest age group. NP T1ρ values and Mean T2 values were similar for cadavers and subjects.	
Wang et al. 2017	MRI indicated that the T1ρ values of nucleus pulposus at L4/5 and L5/S1 were significantly reduced compared with the values of the nucleus pulposus at L1/2, L2/3 and L3/4 (P<0.05), not for T2 values. T1ρ values were significantly different with each Pfirrman grade. All the T1ρ, T2 and ADC values were significantly decreased with the increase of age. T1ρ values remained relatively stable across the age range of 20-45 years and continuously declined after the age of 45 years. T2 values slowly decreased over the age 20-45 and slightly increased at age 45-50. From 50 yrs old all values declined.	Significant difference of T1-ρ between lower and higher lumbar levels. T1ρ is stable age 20-45 and then declines, indicating a stable amount of PG before a decline. T2 values slowly decreased over the age 20-45 and slightly increased at age 45-50, so water content decreases, slightly increases and then from 50 yrs old decreases.
Wei et al. (2022)	Spearman's analysis showed that correlations between T1ρ values of the OPAF, SCEP, ICEP, and NP and modified Pfirrmann grades were significant (P<0.05). T1ρ values of the OPAF, SCEP, and ICEP showed moderate positive correlations with grades with R equal to 0.51, 0.36, and 0.38, respectively. In contrast, the T1ρ of the NP was inversely correlated with grades with an R of −0.94. The T1ρ values of the OAAF and OPAF showed	The negative correlation of T1ρ with the modified Pffirmann in the NP possbily has to do with PG spread into the AF. Furthermore, the positive correlation of T1ρ and Pffirmann in the OPAF has to do with loss of collagen integrity. The higher T1ρ values in the OPAF in with higher Pffirmann grades might be due to spread of PG out of the NP. T1ρ values project PG loss from the NP into the

	positive correlations with ages (R=0.52 and 0.71, respectively), and the T1ρ value of the NP showed an inverse correlation with age (R=−0.76).	AF with age in the group 25-71yrs (linear, not looked per decade).
Yang et al. (2017)	On the longitudinal axis (anterior-posterior direction), the weighted center located posterior to the geometric center in 85.8% of the nuclei whose boundary was clearly identifiable. This finding indicated that the signal intensity was lower at the anterior portion than at the posterior portion in most discs. In intervertebral discs of different Pfirrmann grading, this phenomenon was found in 87.2% of Grade I discs, 89.0% of Grade II discs, and 76.5% of Grade III discs. The prevalence was higher in upper lumbar discs, this phenomenon was still dominant in early degenerative stages of lower lumbar discs (L1/2 91.7%, L2/3 91.5%, L3/4 83.9%, L4/5 86.7%, L5/S1 72.3%).	For age 22-59yrs the weighted center located posterior to the geometric center, which indicated the signal intensity was lower at the anterior portion of the NP, in 85.8% of studied IVDs. Suggesting anterior dominant degeneration for this age group.
Yoon et al. (2016)	There were statistically significant differences in T2 rates between each of the five segmented regions ($p < 0.01$), except for the anterior AF and posterior AF. There were also significant differences in T1ρ rates between all five segmented regions, except for the NP and posterior AF and between the posterior junction and posterior AF. There was a strong correlation between Pfirrmann grade and T2 relaxation times in all	There is strong to moderate correlation between T2 and T1ρ rates and grading of disc degeneration (Pfirrmann score) for the age group 26-84yrs, not considering variation per decade. For T2 the most important regions were the anterior and posterior junction, indicating loss and changes in collagen orientation associated with

	regions except the anterior AF. There were moderate correlations between T1ρ and the posterior junction, NP and anterior junction. There was a significant linear relationship between T2 and T1ρ relaxation rates in all regions.	lamellar disorganization and decreased water and proteoglycan content in the NP. T1ρ imaging is more sensitive to loss of proteoglycan content, resulting in increased relaxation times in the NP where PG content is highest.
Yu et al. (2014)	A significant correlation was observed between degenerative scores and ADC independent of how ADC was obtained. The results suggested a stronger inverse rank correlation between the ADC and the degenerative scores when the ADC value was obtained via sampling the entire IVD compared to that of mid-sagittal ROI only: $\rho = -0.9219$ vs. -0.8655 and -0.9251 vs. -0.9094 from ADC map and ROI-averaged intensities, respectively.	For age 27-62 degenerative scores (Pfirrmann) and ADC had a significant inverse correlation meaning that the ADC decreased as Pfirrmann increased. Larger variation in Pfirrmann grade IV/V discs can be attributed to free moving fluid due to loss of disc integrity.
Zhang et al. (2012)	In the AF, the signal-to-noise ratio (SNR) of each of the 15 independent components of the computed D tensor was poor (less than 10:1), so that maps of DTI indices were unreliable, the results are therefor only presented for the NP. Subject age correlated significantly with MD ($r=-0.72$ with $P<.001$) and moderately with FA ($r=0.45$ with $P<.001$). The FA shows a significant mean increase for the elderly group compared	The increase in FA from young to old is consistent with going from more liquid to a more (thick) fibrous nature of the IVD, progressing more rapidly from age 48. MD decreasing with age is consistent with a reduction of water content and shrinkage of the overall disc volume, to do with PG loss while collagen stays the same. Lumbar

	to the young adult group (P<.001) and the MD demonstrated significant mean decrease for the elderly group compared to the youn adult group (P<.001). Mean FA increased at a greater rate after age 48 years, MD decreased at a greater rate after the age of 48.	discs of lower levels experience more degeneration. All in all there is a significant increase in degradation around age 48.
Zhang et al. (2014)	There was a statistically significant difference in mean ADC values for the five intervertebral disc levels using ANOVA randomized block design ($F = 8.071$, $p < 0.001$). Apparent diffusion coefficient values reduced with decreasing spinal level, with lower ADC values seen in more caudal lumbar discs compared with more cephalad lumbar discs. Pearson's correlation analysis results showed a strong negative association between age and ADC values of all levels (Pearson's correlation coefficient = −0.417, −0.561, −0.650, −0.606, and −0.353 for L1/2, L2/3, L3/4, L4/5, and L5/S1, respectively; all $p < 0.001$).	For age 20-59 ADC values of the IVD decreased with increasing age. As well as that ADC values decreased with increased degeneration (Pffirmann) and lower lumbal discs. Reduction of the ADC values reflect reduced extracellular water motion, indicating loss of disc integrity.
Zobel et al. (2012)	T1ρ value decreased as one proceeded caudally down the spinal column in both sexes. For both sexes, the T1ρ values of the NP were significantly higher compared with the AF values in all levels. The interquartile range shows the wide heterogeneity distribution of T1ρ value, especially for the nondegenerate disc as evaluated according to the Pfirrmann scale. T1ρ	From age 19-25, T1ρ linearly declines from cranial to caudal lumbar levels. T1ρ was significantly lower in women for levels L3-L4 and L4-L5. Nondegenerate discs show a wide range of T1ρ, suggesting heterogeneity of

	value of all the population ranged from 51.7 to 185.5 ms for the 158 discs of grade 1, from 45.0 to 156.9 ms for the 108 discs of grade 2, and from 42.3 to 110.5 ms for the 36 discs of grade 3.	tissue and a 'healthy status'. T1ρ is a good tool to assess early degeneration.

Supplementary file S2:

Table S5 Search Web of Science

#	Search Query	Database	Results	Date Run
1	TI=("intervertebral dis*" OR "vertebral dis*") and Preprint Citation Index (Exclude – Database)	All Databases	10958	Tue Apr 25 2023 13:46:42 GMT+0200
2	AB=("intervertebral dis*" OR "vertebral dis*") and Preprint Citation Index (Exclude – Database)	All Databases	17693	Tue Apr 25 2023 13:56:10 GMT+0200
3	#1 OR #2 and Preprint Citation Index (Exclude – Database)	All Databases	21887	Tue Apr 25 2023 13:56:24 GMT+0200
4	AB=(degenerat* or degradat*) and Preprint Citation Index (Exclude – Database)	All Databases	1152246	Tue Apr 25 2023 13:57:24 GMT+0200
5	TI=(degenerat* or degradat*) and Preprint Citation Index (Exclude – Database)	All Databases	265474	Tue Apr 25 2023 13:57:34 GMT+0200
6	#5 OR #4 and Preprint Citation Index (Exclude – Database)	All Databases	1228758	Tue Apr 25 2023 13:58:23 GMT+0200
7	TI=("magnetic resonance imag*" OR "MR imag*" OR "MRI*" OR "MR tomograph*") and Preprint Citation Index (Exclude – Database)	All Databases	232906	Tue Apr 25 2023 13:59:17 GMT+0200

8	AB=("magnetic resonance imag*" OR "MR imag*" OR "MRI*" OR "MR tomograph*") and Preprint Citation Index (Exclude – Database)	All Databases	547670	Tue Apr 25 2023 13:59:31 GMT+0200
9	#7 OR #8 and Preprint Citation Index (Exclude – Database)	All Databases	624750	Tue Apr 25 2023 13:59:37 GMT+0200
10	#3 AND #6 AND #9 and Preprint Citation Index (Exclude – Database)	All Databases	1769	Tue Apr 25 2023 13:59:50 GMT+0200
11	#3 AND #6 AND #9 and Preprint Citation Index (Exclude – Database) and 2000 or 2023 or 2022 or 2021 or 2020 or 2019 or 2018 or 2017 or 2016 or 2015 or 2014 or 2013 or 2012 or 2011 or 2010 or 2001 or 2002 or 2003 or 2004 or 2005 or 2006 or 2007 or 2008 or 2009 (Publication Years)	All Databases	1626	Tue Apr 25 2023 14:01:44 GMT+0200
12	#3 AND #6 AND #9 and Preprint Citation Index (Exclude – Database) and 2000 or 2023 or 2022 or 2021 or 2020 or 2019 or 2018 or 2017 or 2016 or 2015 or 2014 or 2013 or 2012 or 2011 or 2010 or 2001 or 2002 or 2003 or 2004 or 2005 or 2006 or 2007 or 2008 or 2009 (Publication Years) and Animals (Exclude – MeSH Headings)	All Databases	1201	Tue Apr 25 2023 14:02:24 GMT+0200

Table S6 Search PubMed

Search number	Query	Search Details	Results	Time
8	((((((lumbar intervertebral disc*) OR (lumbar intervertebral disk)) OR (lumbar vertebral disc*)) OR (lumbar vertebral disk) AND (english[Filter])) AND ((((("Intervertebral Disc"[Mesh]) OR (intervertebral disc*)) OR (intervertebral disk)) OR (vertebral disc*)) OR (vertebral disk) AND (english[Filter]))) AND ((((((degenerat*[Title/Abstract]) OR (physiological degenerat*)) OR (physiological age related degenerat*)) OR (age related degenerat*)) OR (intervertebral disc degenerat*)) OR ("Intervertebral Disc	(((("lumbarised"[All Fields] OR "lumbarization"[All Fields] OR "lumbarized"[All Fields] OR "lumbars"[All Fields] OR "lumbosacral region"[MeSH Terms] OR ("lumbosacral"[All Fields] AND "region"[All Fields]) OR "lumbosacral region"[All Fields] OR "lumbar"[All Fields]) AND "intervertebral"[All Fields] AND "disc*"[All Fields]) OR (("lumbarised"[All Fields] OR "lumbarization"[All Fields] OR "lumbarized"[All Fields] OR "lumbars"[All Fields] OR "lumbosacral region"[MeSH Terms] OR ("lumbosacral"[All Fields] AND "region"[All Fields]) OR "lumbosacral region"[All Fields] OR "lumbar"[All Fields]) AND ("Intervertebral Disc"[MeSH Terms] OR ("intervertebral"[All Fields] AND "disc"[All Fields]) OR "Intervertebral Disc"[All Fields] OR ("intervertebral"[All Fields] AND "disk"[All Fields]) OR "intervertebral disk"[All Fields])) OR (("lumbarised"[All Fields] OR "lumbarization"[All Fields] OR "lumbarized"[All Fields] OR "lumbars"[All Fields] OR "lumbosacral region"[MeSH Terms] OR ("lumbosacral"[All Fields] AND "region"[All	1,991	04:37:16

	Degeneration"[Mesh]) AND (english[Filter]))) AND ((Magnetic resonance imaging OR MR imaging OR MRI OR MR tomography) OR ("Magnetic Resonance Imaging"[Mesh]) AND (english[Filter]))	Fields]) OR "lumbosacral region"[All Fields] OR "lumbar"[All Fields]) AND ("spine"[MeSH Terms] OR "spine"[All Fields] OR "vertebral"[All Fields] OR "vertebrals"[All Fields]) AND "disc*"[All Fields]) OR (("lumbarised"[All Fields] OR "lumbarization"[All Fields] OR "lumbarized"[All Fields] OR "lumbars"[All Fields] OR "lumbosacral region"[MeSH Terms] OR ("lumbosacral"[All Fields] AND "region"[All Fields]) OR "lumbosacral region"[All Fields] OR "lumbar"[All Fields]) AND ("spine"[MeSH Terms] OR "spine"[All Fields] OR "vertebral"[All Fields] OR "vertebrals"[All Fields]) AND "disk"[All Fields])) AND "english"[Language] AND (("Intervertebral Disc"[MeSH Terms] OR ("intervertebral"[All Fields] AND "disc*"[All Fields]) OR ("Intervertebral Disc"[MeSH Terms] OR ("intervertebral"[All Fields] AND "disc"[All Fields]) OR "Intervertebral Disc"[All Fields] OR ("intervertebral"[All Fields] AND "disk"[All Fields]) OR "intervertebral disk"[All Fields]) OR (("spine"[MeSH Terms] OR "spine"[All Fields] OR "vertebral"[All Fields] OR "vertebrals"[All Fields]) AND "disc*"[All Fields]) OR (("spine"[MeSH Terms] OR "spine"[All Fields] OR "vertebral"[All Fields] OR "vertebrals"[All Fields]) AND "disk"[All Fields])) AND		

		"english"[Language]) AND (("degenerat*"[Title/Abstract] OR (("physiologic"[All Fields] OR "physiological"[All Fields] OR "physiologically"[All Fields]) AND "degenerat*"[All Fields]) OR (("physiologic"[All Fields] OR "physiological"[All Fields] OR "physiologically"[All Fields]) AND ("agrosyst geosci environ"[Journal] OR "age"[Journal] OR "age omaha"[Journal] OR "age dordr"[Journal] OR "adv genet eng"[Journal] OR "age"[All Fields]) AND ("family"[MeSH Terms] OR "family"[All Fields] OR "relation"[All Fields] OR "relatability"[All Fields] OR "relatable"[All Fields] OR "related"[All Fields] OR "relates"[All Fields] OR "relating"[All Fields] OR "relational"[All Fields] OR "relations"[All Fields]) AND "degenerat*"[All Fields]) OR (("agrosyst geosci environ"[Journal] OR "age"[Journal] OR "age omaha"[Journal] OR "age dordr"[Journal] OR "adv genet eng"[Journal] OR "age"[All Fields]) AND ("family"[MeSH Terms] OR "family"[All Fields] OR "relation"[All Fields] OR "relatability"[All Fields] OR "relatable"[All Fields] OR "related"[All Fields] OR "relates"[All Fields] OR "relating"[All Fields] OR "relational"[All Fields] OR "relations"[All Fields]) AND "degenerat*"[All Fields]) OR (("Intervertebral Disc"[MeSH Terms] OR		

		("intervertebral"[All Fields] AND "disc"[All Fields]) OR "Intervertebral Disc"[All Fields]) AND "degenerat*"[All Fields]) OR "Intervertebral Disc Degeneration"[MeSH Terms]) AND "english"[Language]) AND (("Magnetic Resonance Imaging"[MeSH Terms] OR ("magnetic"[All Fields] AND "resonance"[All Fields] AND "imaging"[All Fields]) OR "Magnetic Resonance Imaging"[All Fields] OR ("Magnetic Resonance Imaging"[MeSH Terms] OR ("magnetic"[All Fields] AND "resonance"[All Fields] AND "imaging"[All Fields]) OR "Magnetic Resonance Imaging"[All Fields] OR ("mr"[All Fields] AND "imaging"[All Fields]) OR "mr imaging"[All Fields]) OR ("Magnetic Resonance Imaging"[MeSH Terms] OR ("magnetic"[All Fields] AND "resonance"[All Fields] AND "imaging"[All Fields]) OR "Magnetic Resonance Imaging"[All Fields] OR "mri"[All Fields]) OR ("Magnetic Resonance Imaging"[MeSH Terms] OR ("magnetic"[All Fields] AND "resonance"[All Fields] AND "imaging"[All Fields]) OR "Magnetic Resonance Imaging"[All Fields] OR ("mr"[All Fields] AND "tomography"[All Fields]) OR "mr tomography"[All Fields]) OR "Magnetic Resonance Imaging"[MeSH Terms]) AND		

		"english"[Language])) AND ((humans[Filter]) AND (english[Filter]) AND (2000:2023[pdat]))		
7	((((((lumbar intervertebral disc*) OR (lumbar intervertebral disk)) OR (lumbar vertebral disc*)) OR (lumbar vertebral disk) AND (english[Filter])) AND (((((("Intervertebral Disc"[Mesh]) OR (intervertebral disc*)) OR (intervertebral disk)) OR (vertebral disc*)) OR (vertebral disk) AND (english[Filter]))) AND ((((((degenerat*[Title/Abstract]) OR (physiological degenerat*)) OR (physiological age related degenerat*)) OR (age related degenerat*)) OR (intervertebral disc degenerat*)) OR ("Intervertebral Disc Degeneration"[Mesh]) AND	(((("lumbarised"[All Fields] OR "lumbarization"[All Fields] OR "lumbarized"[All Fields] OR "lumbars"[All Fields] OR "lumbosacral region"[MeSH Terms] OR ("lumbosacral"[All Fields] AND "region"[All Fields]) OR "lumbosacral region"[All Fields] OR "lumbar"[All Fields]) AND "intervertebral"[All Fields] AND "disc*"[All Fields]) OR (("lumbarised"[All Fields] OR "lumbarization"[All Fields] OR "lumbarized"[All Fields] OR "lumbars"[All Fields] OR "lumbosacral region"[MeSH Terms] OR ("lumbosacral"[All Fields] AND "region"[All Fields]) OR "lumbosacral region"[All Fields] OR "lumbar"[All Fields]) AND ("Intervertebral Disc"[MeSH Terms] OR ("intervertebral"[All Fields] AND "disc"[All Fields]) OR "Intervertebral Disc"[All Fields] OR ("intervertebral"[All Fields] AND "disk"[All Fields]) OR "intervertebral disk"[All Fields])) OR (("lumbarised"[All Fields] OR "lumbarization"[All Fields] OR "lumbarized"[All Fields] OR "lumbars"[All Fields] OR "lumbosacral region"[MeSH Terms] OR ("lumbosacral"[All Fields] AND "region"[All Fields]) OR "lumbosacral region"[All Fields] OR "lumbar"[All Fields]) AND	2,502	04:37:09

	(english[Filter]))) AND ((Magnetic resonance imaging OR MR imaging OR MRI OR MR tomography) OR ("Magnetic Resonance Imaging"[Mesh]) AND (english[Filter]))	("spine"[MeSH Terms] OR "spine"[All Fields] OR "vertebral"[All Fields] OR "vertebrals"[All Fields]) AND "disc*"[All Fields]) OR (("lumbarised"[All Fields] OR "lumbarization"[All Fields] OR "lumbarized"[All Fields] OR "lumbars"[All Fields] OR "lumbosacral region"[MeSH Terms] OR ("lumbosacral"[All Fields] AND "region"[All Fields]) OR "lumbosacral region"[All Fields] OR "lumbar"[All Fields]) AND ("spine"[MeSH Terms] OR "spine"[All Fields] OR "vertebral"[All Fields] OR "vertebrals"[All Fields]) AND "disk"[All Fields])) AND "english"[Language] AND (("Intervertebral Disc"[MeSH Terms] OR ("intervertebral"[All Fields] AND "disc*"[All Fields]) OR ("Intervertebral Disc"[MeSH Terms] OR ("intervertebral"[All Fields] AND "disc"[All Fields]) OR "Intervertebral Disc"[All Fields] OR ("intervertebral"[All Fields] AND "disk"[All Fields]) OR "intervertebral disk"[All Fields]) OR (("spine"[MeSH Terms] OR "spine"[All Fields] OR "vertebral"[All Fields] OR "vertebrals"[All Fields]) AND "disc*"[All Fields]) OR (("spine"[MeSH Terms] OR "spine"[All Fields] OR "vertebral"[All Fields] OR "vertebrals"[All Fields]) AND "disk"[All Fields])) AND "english"[Language]) AND (("degenerat*"[Title/Abstract] OR		

		(("physiologic"[All Fields] OR "physiological"[All Fields] OR "physiologically"[All Fields]) AND "degenerat*"[All Fields]) OR (("physiologic"[All Fields] OR "physiological"[All Fields] OR "physiologically"[All Fields]) AND ("agrosyst geosci environ"[Journal] OR "age"[Journal] OR "age omaha"[Journal] OR "age dordr"[Journal] OR "adv genet eng"[Journal] OR "age"[All Fields]) AND ("family"[MeSH Terms] OR "family"[All Fields] OR "relation"[All Fields] OR "relatability"[All Fields] OR "relatable"[All Fields] OR "related"[All Fields] OR "relates"[All Fields] OR "relating"[All Fields] OR "relational"[All Fields] OR "relations"[All Fields]) AND "degenerat*"[All Fields]) OR (("agrosyst geosci environ"[Journal] OR "age"[Journal] OR "age omaha"[Journal] OR "age dordr"[Journal] OR "adv genet eng"[Journal] OR "age"[All Fields]) AND ("family"[MeSH Terms] OR "family"[All Fields] OR "relation"[All Fields] OR "relatability"[All Fields] OR "relatable"[All Fields] OR "related"[All Fields] OR "relates"[All Fields] OR "relating"[All Fields] OR "relational"[All Fields] OR "relations"[All Fields]) AND "degenerat*"[All Fields]) OR (("Intervertebral Disc"[MeSH Terms] OR ("intervertebral"[All Fields] AND "disc"[All Fields]) OR "Intervertebral		

		Disc"[All Fields]) AND "degenerat*"[All Fields]) OR "Intervertebral Disc Degeneration"[MeSH Terms]) AND "english"[Language]) AND (("Magnetic Resonance Imaging"[MeSH Terms] OR ("magnetic"[All Fields] AND "resonance"[All Fields] AND "imaging"[All Fields]) OR "Magnetic Resonance Imaging"[All Fields] OR ("Magnetic Resonance Imaging"[MeSH Terms] OR ("magnetic"[All Fields] AND "resonance"[All Fields] AND "imaging"[All Fields]) OR "Magnetic Resonance Imaging"[All Fields] OR ("mr"[All Fields] AND "imaging"[All Fields]) OR "mr imaging"[All Fields]) OR ("Magnetic Resonance Imaging"[MeSH Terms] OR ("magnetic"[All Fields] AND "resonance"[All Fields] AND "imaging"[All Fields]) OR "Magnetic Resonance Imaging"[All Fields] OR "mri"[All Fields]) OR ("Magnetic Resonance Imaging"[MeSH Terms] OR ("magnetic"[All Fields] AND "resonance"[All Fields] AND "imaging"[All Fields]) OR "Magnetic Resonance Imaging"[All Fields] OR ("mr"[All Fields] AND "tomography"[All Fields]) OR "mr tomography"[All Fields]) OR "Magnetic Resonance Imaging"[MeSH Terms]) AND "english"[Language])) AND ((english[Filter]) AND (2000:2023[pdat]))		

6	((((((lumbar intervertebral disc*) OR (lumbar intervertebral disk)) OR (lumbar vertebral disc*)) OR (lumbar vertebral disk) AND (english[Filter])) AND ((((("Intervertebral Disc"[Mesh]) OR (intervertebral disc*)) OR (intervertebral disk)) OR (vertebral disc*)) OR (vertebral disk) AND (english[Filter]))) AND ((((((degenerat*[Title/Abstract]) OR (physiological degenerat*)) OR (physiological age related degenerat*)) OR (age related degenerat*)) OR (intervertebral disc degenerat*)) OR ("Intervertebral Disc Degeneration"[Mesh]) AND (english[Filter]))) AND ((Magnetic resonance imaging OR MR imaging OR	(((("lumbarised"[All Fields] OR "lumbarization"[All Fields] OR "lumbarized"[All Fields] OR "lumbars"[All Fields] OR "lumbosacral region"[MeSH Terms] OR ("lumbosacral"[All Fields] AND "region"[All Fields]) OR "lumbosacral region"[All Fields] OR "lumbar"[All Fields]) AND "intervertebral"[All Fields] AND "disc*"[All Fields]) OR (("lumbarised"[All Fields] OR "lumbarization"[All Fields] OR "lumbarized"[All Fields] OR "lumbars"[All Fields] OR "lumbosacral region"[MeSH Terms] OR ("lumbosacral"[All Fields] AND "region"[All Fields]) OR "lumbosacral region"[All Fields] OR "lumbar"[All Fields]) AND ("Intervertebral Disc"[MeSH Terms] OR ("intervertebral"[All Fields] AND "disc"[All Fields]) OR "Intervertebral Disc"[All Fields] OR ("intervertebral"[All Fields] AND "disk"[All Fields]) OR "intervertebral disk"[All Fields])) OR (("lumbarised"[All Fields] OR "lumbarization"[All Fields] OR "lumbarized"[All Fields] OR "lumbars"[All Fields] OR "lumbosacral region"[MeSH Terms] OR ("lumbosacral"[All Fields] AND "region"[All Fields]) OR "lumbosacral region"[All Fields] OR "lumbar"[All Fields]) AND ("spine"[MeSH Terms] OR "spine"[All Fields] OR "vertebral"[All Fields] OR "vertebrals"[All Fields]) AND "disc*"[All Fields]) OR	2,752	04:36:55

	MRI OR MR tomography) OR ("Magnetic Resonance Imaging"[Mesh]) AND (english[Filter]))	(("lumbarised"[All Fields] OR "lumbarization"[All Fields] OR "lumbarized"[All Fields] OR "lumbars"[All Fields] OR "lumbosacral region"[MeSH Terms] OR ("lumbosacral"[All Fields] AND "region"[All Fields]) OR "lumbosacral region"[All Fields] OR "lumbar"[All Fields]) AND ("spine"[MeSH Terms] OR "spine"[All Fields] OR "vertebral"[All Fields] OR "vertebrals"[All Fields]) AND "disk"[All Fields])) AND "english"[Language] AND (("Intervertebral Disc"[MeSH Terms] OR ("intervertebral"[All Fields] AND "disc*"[All Fields]) OR ("Intervertebral Disc"[MeSH Terms] OR ("intervertebral"[All Fields] AND "disc"[All Fields]) OR "Intervertebral Disc"[All Fields] OR ("intervertebral"[All Fields] AND "disk"[All Fields]) OR "intervertebral disk"[All Fields]) OR (("spine"[MeSH Terms] OR "spine"[All Fields] OR "vertebral"[All Fields] OR "vertebrals"[All Fields]) AND "disc*"[All Fields]) OR (("spine"[MeSH Terms] OR "spine"[All Fields] OR "vertebral"[All Fields] OR "vertebrals"[All Fields]) AND "disk"[All Fields])) AND "english"[Language]) AND (("degenerat*"[Title/Abstract] OR (("physiologic"[All Fields] OR "physiological"[All Fields] OR "physiologically"[All Fields]) AND "degenerat*"[All Fields]) OR		

		(("physiologic"[All Fields] OR "physiological"[All Fields] OR "physiologically"[All Fields]) AND ("agrosyst geosci environ"[Journal] OR "age"[Journal] OR "age omaha"[Journal] OR "age dordr"[Journal] OR "adv genet eng"[Journal] OR "age"[All Fields]) AND ("family"[MeSH Terms] OR "family"[All Fields] OR "relation"[All Fields] OR "relatability"[All Fields] OR "relatable"[All Fields] OR "related"[All Fields] OR "relates"[All Fields] OR "relating"[All Fields] OR "relational"[All Fields] OR "relations"[All Fields]) AND "degenerat*"[All Fields]) OR (("agrosyst geosci environ"[Journal] OR "age"[Journal] OR "age omaha"[Journal] OR "age dordr"[Journal] OR "adv genet eng"[Journal] OR "age"[All Fields]) AND ("family"[MeSH Terms] OR "family"[All Fields] OR "relation"[All Fields] OR "relatability"[All Fields] OR "relatable"[All Fields] OR "related"[All Fields] OR "relates"[All Fields] OR "relating"[All Fields] OR "relational"[All Fields] OR "relations"[All Fields]) AND "degenerat*"[All Fields]) OR (("Intervertebral Disc"[MeSH Terms] OR ("intervertebral"[All Fields] AND "disc"[All Fields]) OR "Intervertebral Disc"[All Fields]) AND "degenerat*"[All Fields]) OR "Intervertebral Disc Degeneration"[MeSH Terms]) AND "english"[Language]) AND		

		(("Magnetic Resonance Imaging"[MeSH Terms] OR ("magnetic"[All Fields] AND "resonance"[All Fields] AND "imaging"[All Fields]) OR "Magnetic Resonance Imaging"[All Fields] OR ("Magnetic Resonance Imaging"[MeSH Terms] OR ("magnetic"[All Fields] AND "resonance"[All Fields] AND "imaging"[All Fields]) OR "Magnetic Resonance Imaging"[All Fields] OR ("mr"[All Fields] AND "imaging"[All Fields]) OR "mr imaging"[All Fields]) OR ("Magnetic Resonance Imaging"[MeSH Terms] OR ("magnetic"[All Fields] AND "resonance"[All Fields] AND "imaging"[All Fields]) OR "Magnetic Resonance Imaging"[All Fields] OR "mri"[All Fields]) OR ("Magnetic Resonance Imaging"[MeSH Terms] OR ("magnetic"[All Fields] AND "resonance"[All Fields] AND "imaging"[All Fields]) OR "Magnetic Resonance Imaging"[All Fields] OR ("mr"[All Fields] AND "tomography"[All Fields]) OR "mr tomography"[All Fields]) OR "Magnetic Resonance Imaging"[MeSH Terms]) AND "english"[Language])) AND (english[Filter])		
5	(Magnetic resonance imaging OR MR imaging OR MRI OR MR tomography)	("Magnetic Resonance Imaging"[MeSH Terms] OR ("magnetic"[All Fields] AND "resonance"[All Fields] AND "imaging"[All Fields]) OR "Magnetic Resonance Imaging"[All Fields] OR ("Magnetic Resonance	697,919	04:36:37

	OR ("Magnetic Resonance Imaging"[Mesh])	Imaging"[MeSH Terms] OR ("magnetic"[All Fields] AND "resonance"[All Fields] AND "imaging"[All Fields]) OR "Magnetic Resonance Imaging"[All Fields] OR ("mr"[All Fields] AND "imaging"[All Fields]) OR "mr imaging"[All Fields]) OR ("Magnetic Resonance Imaging"[MeSH Terms] OR ("magnetic"[All Fields] AND "resonance"[All Fields] AND "imaging"[All Fields]) OR "Magnetic Resonance Imaging"[All Fields] OR "mri"[All Fields]) OR ("Magnetic Resonance Imaging"[MeSH Terms] OR ("magnetic"[All Fields] AND "resonance"[All Fields] AND "imaging"[All Fields]) OR "Magnetic Resonance Imaging"[All Fields] OR ("mr"[All Fields] AND "tomography"[All Fields]) OR "mr tomography"[All Fields]) OR "Magnetic Resonance Imaging"[MeSH Terms]) AND (english[Filter])		
4	(((((degenerat*[Title/Abstract]) OR (physiological degenerat*)) OR (physiological age related degenerat*)) OR (age related degenerat*)) OR (intervertebral disc degenerat*)) OR ("Intervertebral Disc Degeneration"[Mesh])	("degenerat*"[Title/Abstract] OR (("physiologic"[All Fields] OR "physiological"[All Fields] OR "physiologically"[All Fields]) AND "degenerat*"[All Fields]) OR (("physiologic"[All Fields] OR "physiological"[All Fields] OR "physiologically"[All Fields]) AND ("agrosyst geosci environ"[Journal] OR "age"[Journal] OR "age omaha"[Journal] OR "age dordr"[Journal] OR "adv genet eng"[Journal] OR "age"[All Fields]) AND ("family"[MeSH Terms] OR "family"[All Fields]	230,08	04:36:20

		OR "relation"[All Fields] OR "relatability"[All Fields] OR "relatable"[All Fields] OR "related"[All Fields] OR "relates"[All Fields] OR "relating"[All Fields] OR "relational"[All Fields] OR "relations"[All Fields]) AND "degenerat*"[All Fields]) OR (("agrosyst geosci environ"[Journal] OR "age"[Journal] OR "age omaha"[Journal] OR "age dordr"[Journal] OR "adv genet eng"[Journal] OR "age"[All Fields]) AND ("family"[MeSH Terms] OR "family"[All Fields] OR "relation"[All Fields] OR "relatability"[All Fields] OR "relatable"[All Fields] OR "related"[All Fields] OR "relates"[All Fields] OR "relating"[All Fields] OR "relational"[All Fields] OR "relations"[All Fields]) AND "degenerat*"[All Fields]) OR (("intervertebral disc"[MeSH Terms] OR ("intervertebral"[All Fields] AND "disc"[All Fields]) OR "intervertebral disc"[All Fields]) AND "degenerat*"[All Fields]) OR "Intervertebral Disc Degeneration"[MeSH Terms]) AND (english[Filter])		
3	((((degenerat*[Title/Abstract]) OR (physiological degenerat*)) OR (physiological age related degenerat*)) OR (age related degenerat*)) OR	("degenerat*"[Title/Abstract] OR (("physiologic"[All Fields] OR "physiological"[All Fields] OR "physiologically"[All Fields]) AND "degenerat*"[All Fields]) OR (("physiologic"[All Fields] OR "physiological"[All Fields] OR "physiologically"[All Fields]) AND	230,08	04:34:29

	("Intervertebral Disc Degeneration"[Mesh])) OR (intervertebral disc degenerat*)))	("agrosyst geosci environ"[Journal] OR "age"[Journal] OR "age omaha"[Journal] OR "age dordr"[Journal] OR "adv genet eng"[Journal] OR "age"[All Fields]) AND ("family"[MeSH Terms] OR "family"[All Fields] OR "relation"[All Fields] OR "relatability"[All Fields] OR "relatable"[All Fields] OR "related"[All Fields] OR "relates"[All Fields] OR "relating"[All Fields] OR "relational"[All Fields] OR "relations"[All Fields]) AND "degenerat*"[All Fields]) OR (("agrosyst geosci environ"[Journal] OR "age"[Journal] OR "age omaha"[Journal] OR "age dordr"[Journal] OR "adv genet eng"[Journal] OR "age"[All Fields]) AND ("family"[MeSH Terms] OR "family"[All Fields] OR "relation"[All Fields] OR "relatability"[All Fields] OR "relatable"[All Fields] OR "related"[All Fields] OR "relates"[All Fields] OR "relating"[All Fields] OR "relational"[All Fields] OR "relations"[All Fields]) AND "degenerat*"[All Fields]) OR "Intervertebral Disc Degeneration"[MeSH Terms] OR (("intervertebral disc"[MeSH Terms] OR ("intervertebral"[All Fields] AND "disc"[All Fields]) OR "intervertebral disc"[All Fields]) AND "degenerat*"[All Fields])) AND (english[Filter])		

2	(((("Intervertebral Disc"[Mesh]) OR (intervertebral disc*)) OR (intervertebral disk)) OR (vertebral disc*)) OR (vertebral disk)	("Intervertebral Disc"[MeSH Terms] OR ("intervertebral"[All Fields] AND "disc*"[All Fields]) OR ("Intervertebral Disc"[MeSH Terms] OR ("intervertebral"[All Fields] AND "disc"[All Fields]) OR "Intervertebral Disc"[All Fields] OR ("intervertebral"[All Fields] AND "disk"[All Fields]) OR "intervertebral disk"[All Fields]) OR (("spine"[MeSH Terms] OR "spine"[All Fields] OR "vertebral"[All Fields] OR "vertebrals"[All Fields]) AND "disc*"[All Fields]) OR (("spine"[MeSH Terms] OR "spine"[All Fields] OR "vertebral"[All Fields] OR "vertebrals"[All Fields]) AND "disk"[All Fields])) AND (english[Filter])	78,805	04:32:07
1	(((lumbar intervertebral disc*) OR (lumbar intervertebral disk)) OR (lumbar vertebral disc*)) OR (lumbar vertebral disk)	((("lumbarised"[All Fields] OR "lumbarization"[All Fields] OR "lumbarized"[All Fields] OR "lumbars"[All Fields] OR "lumbosacral region"[MeSH Terms] OR ("lumbosacral"[All Fields] AND "region"[All Fields]) OR "lumbosacral region"[All Fields] OR "lumbar"[All Fields]) AND "intervertebral"[All Fields] AND "disc*"[All Fields]) OR (("lumbarised"[All Fields] OR "lumbarization"[All Fields] OR "lumbarized"[All Fields] OR "lumbars"[All Fields] OR "lumbosacral region"[MeSH Terms] OR ("lumbosacral"[All Fields] AND "region"[All Fields]) OR "lumbosacral region"[All Fields] OR "lumbar"[All Fields]) AND ("intervertebral	31,606	04:31:02

		disc"[MeSH Terms] OR ("intervertebral"[All Fields] AND "disc"[All Fields]) OR "intervertebral disc"[All Fields] OR ("intervertebral"[All Fields] AND "disk"[All Fields]) OR "intervertebral disk"[All Fields])) OR (("lumbarised"[All Fields] OR "lumbarization"[All Fields] OR "lumbarized"[All Fields] OR "lumbars"[All Fields] OR "lumbosacral region"[MeSH Terms] OR ("lumbosacral"[All Fields] AND "region"[All Fields]) OR "lumbosacral region"[All Fields] OR "lumbar"[All Fields]) AND ("spine"[MeSH Terms] OR "spine"[All Fields] OR "vertebral"[All Fields] OR "vertebrals"[All Fields]) AND "disc*"[All Fields]) OR (("lumbarised"[All Fields] OR "lumbarization"[All Fields] OR "lumbarized"[All Fields] OR "lumbars"[All Fields] OR "lumbosacral region"[MeSH Terms] OR ("lumbosacral"[All Fields] AND "region"[All Fields]) OR "lumbosacral region"[All Fields] OR "lumbar"[All Fields]) AND ("spine"[MeSH Terms] OR "spine"[All Fields] OR "vertebral"[All Fields] OR "vertebrals"[All Fields]) AND "disk"[All Fields])) AND (english[Filter])		

Table S7 Search Embase

#	Searches	Results

1	*intervertebral disk/ or "intervertebral dis*".ti,ab. or "vertebral dis*".ti,ab.	24102
2	*intervertebral disk degeneration/ or "degradat*".ti,ab. or "degenerat*".ti,ab.	757002
3	*nuclear magnetic resonance imaging/ or "magnetic resonance imag*".ti,ab. or "MR imag*".ti,ab. or MRI.ti,ab. or MRIs.ti,ab. or "MRI's".ti,ab. or "MR tomograph*".ti,ab.	799286
4	exp animal/ not human/	5256369
5	(1 and 2 and 3) not (exp animal/ not human/)	1938
6	*intervertebral disk/	5610
7	5 and 2000:2024.(sa_year).	1768

Table S8 Search Cochrane

ID	Search
#1	MeSH descriptor: [Intervertebral Disc] explode all trees
#2	(intervertebral dis*) OR (vertebral dis*) (Word variations have been searched)
#3	#1 OR #2
#4	(lumbar intervertebral dis*) OR (lumbar vertebral dis*) OR (lumbar dis*) (Word variations have been searched)
#5	MeSH descriptor: [Intervertebral Disc Degeneration] explode all trees
#6	(degenerat*):ti,ab,kw OR (age related degenerat*) OR (physiological degenerat*) OR (physiological age related degenerat*) OR (intervertebral disc degenerat*) (Word variations have been searched)
#7	#5 OR #6
#8	MeSH descriptor: [Magnetic Resonance Imaging] explode all trees
#9	(MRI) OR (Magnetic resonance imaging) OR (Magnetic resonance scan*) OR ("magnetic resonance tomography") (Word variations have been searched)
#10	#3 AND #4 AND #7 AND #9
#11	#4 AND #7 AND #9
#12	#3 AND #7 AND #9
#13	#3 AND #4 AND #7
